# Analysis of Compensatory Movements Using a Supernumerary Robotic Hand for Upper Limb Assistance

**DOI:** 10.3389/frobt.2020.587759

**Published:** 2020-12-17

**Authors:** Martina Rossero, Andrea S. Ciullo, Giorgio Grioli, Manuel G. Catalano, Antonio Bicchi

**Affiliations:** ^1^Soft Robotics for Human Cooperation and Rehabilitation, Italian Institute of Technology, Genoa, Italy; ^2^Centro “E. Piaggio” and Dipartimento di Ingegneria dell'Informazione, University of Pisa, Pisa, Italy

**Keywords:** compensatory movements, kinematic analysis, soft robotics, supernumerary robotic limbs, robotic assistance

## Abstract

Recently, extratheses, aka Supernumerary Robotic Limbs (SRLs), are emerging as a new trend in the field of assistive and rehabilitation devices. We proposed the SoftHand X, a system composed of an anthropomorphic soft hand extrathesis, with a gravity support boom and a control interface for the patient. In preliminary tests, the system exhibited a positive outlook toward assisting impaired people during daily life activities and fighting learned-non-use of the impaired arm. However, similar to many robot-aided therapies, the use of the system may induce side effects that can be detrimental and worsen patients' conditions. One of the most common is the onset of alternative grasping strategies and compensatory movements, which clinicians absolutely need to counter in physical therapy. Before embarking in systematic experimentation with the SoftHand X on patients, it is essential that the system is demonstrated not to lead to an increase of compensation habits. This paper provides a detailed description of the compensatory movements performed by healthy subjects using the SoftHand X. Eleven right-handed healthy subjects were involved within an experimental protocol in which kinematic data of the upper body and EMG signals of the arm were acquired. Each subject executed tasks with and without the robotic system, considering this last situation as reference of optimal behavior. A comparison between two different configurations of the robotic hand was performed to understand if this aspect may affect the compensatory movements. Results demonstrated that the use of the apparatus reduces the range of motion of the wrist, elbow and shoulder, while it increases the range of the trunk and head movements. On the other hand, EMG analysis indicated that muscle activation was very similar among all the conditions. Results obtained suggest that the system may be used as assistive device without causing an over-use of the arm joints, and opens the way to clinical trials with patients.

## 1. Introduction

One of the main symptoms of neuro-muscular diseases consists of partial or total loss of motor functions, such as walking or manipulating objects (Wade, 1992; Mozaffarian et al., 2015). Considering the upper extremities, the functional reduction of the hand-arm may drastically compromise the independence of the subject, hampering the ability in performing many Activities of Daily Living (ADL) (Mondiale de la Santé and Organization, 2001). In the last decades, to flank standard medical therapy, many robotic devices have been proposed in an attempt to counteract these issues and promote motor recovery (Maciejasz et al., 2014). Recently, a new trend is emerging in the robotic field: Supernumerary Robotics Limbs (SRLs). Initially developed to improve the user's ergonomics and capacity in industrial applications (Llorens-Bonilla et al., 2012; Parietti and Asada, 2017; Ciullo et al., 2018a), they consist of additional artificial limbs that can perform tasks in close coordination with the subject wearing them. Their clinical use was pioneered by Hussain et al. (2016) where an additional robotic finger (the Sixth finger) was used for compensating hand missing abilities in chronic stroke subjects. Another device for clinical application can be found in Ciullo et al. (2020) where the SoftHand X (SHX) system is described and tested with ten post-stroke chronic subjects. It consists of an anthropomorphic artificial hand, a passive gravity compensator and an input interface used by the subject to control the device. Results showed that this system significantly improved the performances of the patients in the proposed tasks and, more in general, their autonomy in ADL. Nine out of ten patients were able to perform the whole task proposed and asserted that they would use the system in their daily life. However, it must be noted that the use of such devices may induce some side effects that can be harmful, limiting the recovery of normal movement patterns or even promoting pathological conditions, such as spasticity (Ada et al., 1994). In Ciullo et al. (2020), spasticity before and after use was measured by the Modified Ashworth Scale (MAS). Seven patients exhibited a reduction of the MAS (no statistical relevance was proven, however). Another issue can be the onset of some compensatory movements in which alternative muscles and motor strategies are used to complete a task (Levin et al., 2009). Due to the impairment, most of the patients are used to run into these strategies, so it is essential that robotic systems do not worsen this situation. In literature, some discussions and analysis have been already proposed to evaluate compensatory movements in post-stroke subjects (Cirstea and Levin, 2000; Roby-Brami et al., 2003; Michaelsen et al., 2004). These compensatory strategies most prominently involve the use of the trunk, the shoulder or proximal residual muscles capabilities to perform the requested tasks (Metzger et al., 2012; Hussaini et al., 2017). Similar investigations have been conducted also for upper limb prosthesis users (Carey et al., 2008; Metzger et al., 2012; Major et al., 2014). In Carey et al. (2008) the compensatory movements of transradial prosthesis users without wrist motion have been compared to that of non-amputees under an unrestricted and restricted forearm rotation conditions. In tasks requiring a larger forearm rotation and wrist flexion, persons with transradial amputation compensated predominantly with movements of the torso side bending toward the affected side and with elbow flexion. In tasks not requiring as much forearm rotation, such as drinking from a cup, the location of compensation was not determined. This study has been extended including transhumeral prosthesis and body-powered devices users (Metzger et al., 2012) confirming the presence of compensatory movement for the trunk and proximal upper limb.

**Figure 1:**
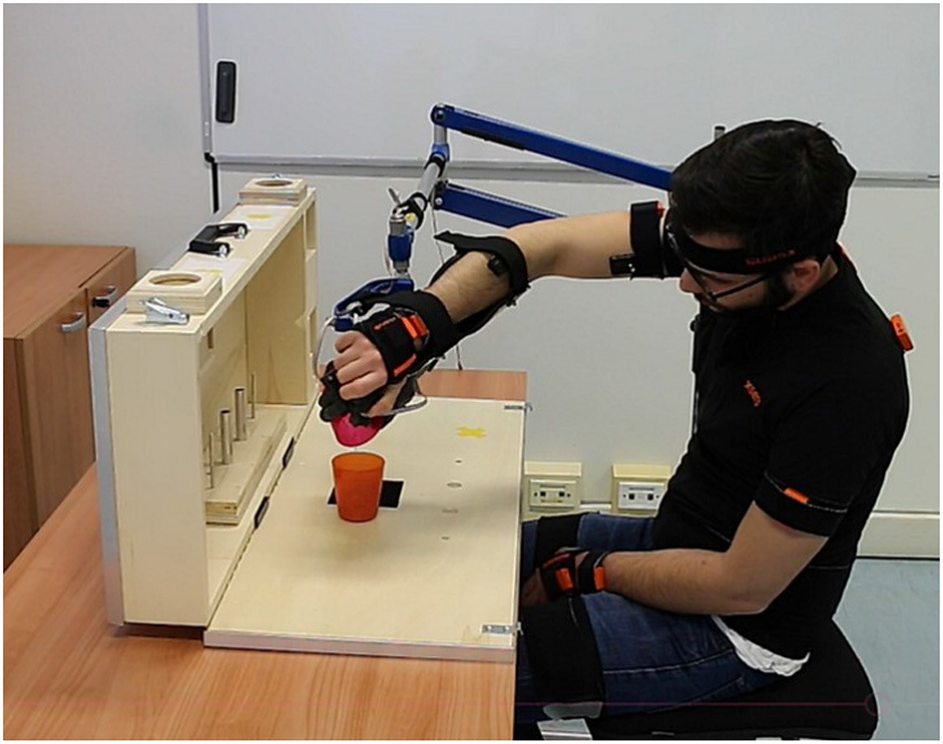
Example of head and trunk compensatory movements performed by a subject during the execution of a task of the ARAT test (pouring task).

SRLs, due to their encumbrance and position with respect to the natural limbs, may have a higher predisposition to induce compensatory movements. No work has been conducted to analyze this compensation but it is important to quantify them before approaching clinical trials. This work aims at providing a detailed description of these compensatory movements (see an example in Figure 1) arising while using the SHX system to assist upper limb motion during the execution of some exemplary tasks.

Inspired by the methodologies adopted in the previous investigations cited, this work compares the performance of eleven right-handed healthy subjects executing grasping tasks with and without the robotic system. During the experiment, kinematic data of the upper body and EMG signals of the arm have been acquired. Results show a reduction on the arm joints Ranges of Motion, compensated by trunk and head movements. This suggests the possibility of using the SHX to assist impaired subject without over-stressing the impaired arm. However, the increase in the use of trunk and head can be harmful in the long term so a new version of the human arm interface will be developed to counteract these effects.

## 2. Materials and Methods

Eleven right-handed healthy subjects (five males, six females, mean age 27) were involved within an experimental procedure approved by the Institutional Review Board of the University of Pisa, in accordance with the Declaration of Helsinki. Each subject signed the inform consent before starting the experimental session.

### 2.1. Experimental Setup

**Figure 2:**
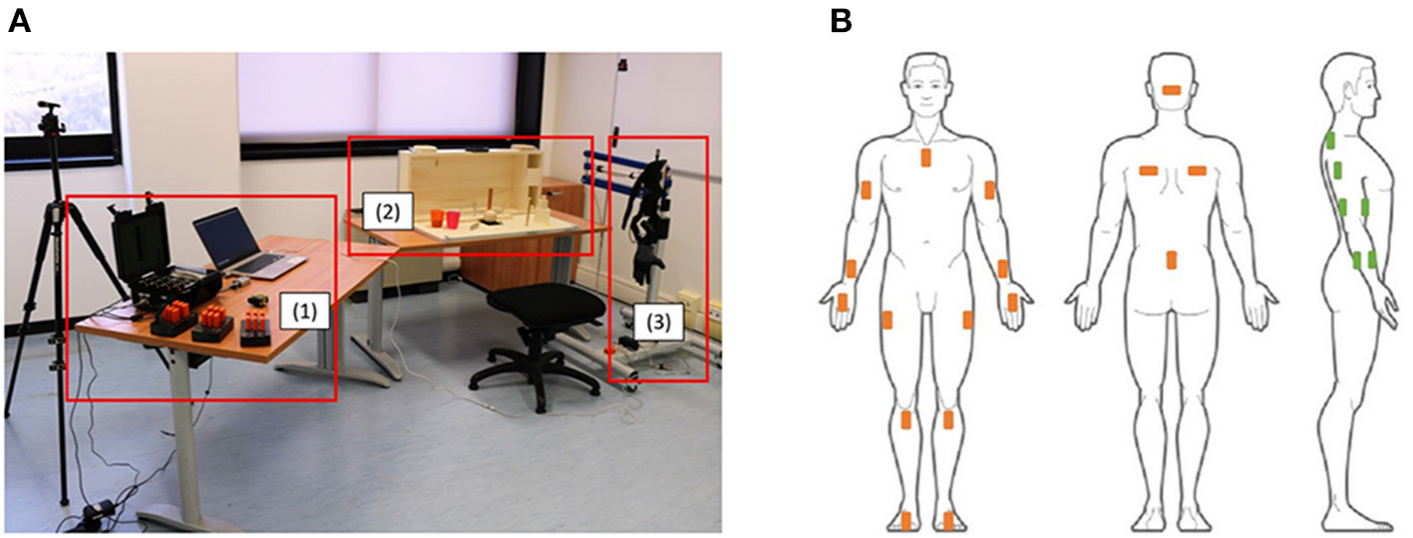
**(A)** Shows a view of the experimental setup where (1) shows the data acquisition systems, (2) shows the ARAT test and (3) shows the SHX system. **(B)** Shows the positioning of the Xsens sensors (in orange) and of the EMG electrodes on the right arm (in green).

Kinematic data of the upper body and EMG signals of the arm were acquired during tasks^1^ using two acquisition systems, as shown in Figure 2A.

The Xsens MVN system, composed of 17 IMU sensors placed on the subject body (as shown in Figure 2B), was used to obtain kinematic data recorded with a sampling frequency of 60 Hz. For each body segment, the data acquired were the position, the orientation, the velocity, and the acceleration. Moreover, the joint angles of the shoulder, the elbow, the wrist, the trunk, and the neck were measured by the system.

A Trigno Delsys wireless system was used to record the electromyographic signal (sampling frequency 2 kHz) of the following muscles of the right arm: trapezius, lateral deltoid, biceps, triceps lateral head, ulnar flexor and extensor. Additionally, the commanded and the real pose of the robotic hand were registered. To obtain a time consistent description of the movements, all the data were synchronized with a unique custom C++ interface.

**Figure 3:**
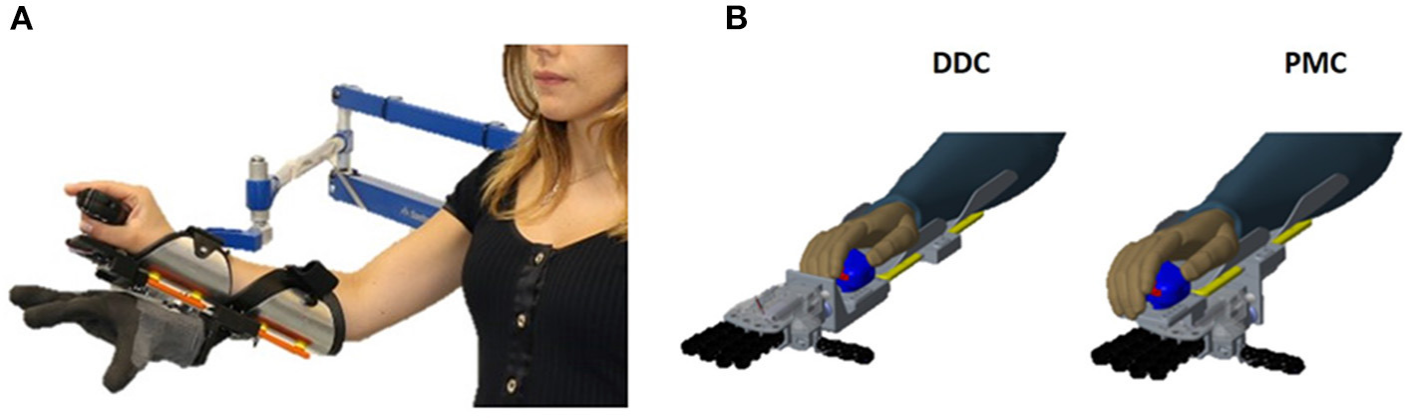
**(A)** Shows the SoftHand X system used by the subjects during the experiments. **(B)** Shows the two configuration tested. The first has the robotic hand in front of the natural one, aligned with the user's arm (Dorsal Distal Central, DDC), while the second has the robotic hand below the natural one (Palmar Middle Central, PMC).

The SHX system used (shown in Figure 3A) is a modular robotic system for the upper limb support with anthropomorphic characteristics and inspired by neuroscientific theories of motor control (Santello et al., 1998; Della Santina et al., 2017). It is designed to be used for clinical investigation, either for the rehabilitation and for the assistance of patients with neuro-muscular diseases (e.g., stroke patient) or of elder people with weak muscles in the upper limb. It is composed of single separated sub-parts that are conveniently assembled for the user's needs. In particular, it consists of an end effector that is a Pisa/IIT SoftHand implementing the function of the human hand and a passive gravity compensator. These two parts are integrated by a human-arm interface with a wrist-like structure designed as additional component with the basic function of connecting them and allowing the robotic hand to be used as an extra thesis [from which the name SoftHand eXtrathesis (SoftHand X)]. An input interface, connected to the SHX thanks to a workstation, is used by the subject to control the robotic hand. In this study, the robotic hand is activated with a hand-held handle controlled by the right natural hand of the subject. This could be considered as a limitation for the supernumerary system since the natural hand is not free to move. However, the system is thought to be used by impaired subject that can use the additional hand to recover the lost hand functions and at the same time train the movement of the natural one. Moreover, other input interfaces have been designed to control the opening and closing of the SoftHand with feet and facial muscles to leave all the hand free to be used (Ciullo et al., 2020). To investigate if the position of the robotic hand could influence the compensatory movements exploited by the subjects, two different configurations have been tested. Such configurations are the result of a previous optimization study, where the manipulability and workspace of the system where analyzed (Ciullo et al., 2018b). The first configuration has the robotic hand in front of the natural one, aligned with the user's arm (Dorsal Distal Central, DDC), while the second has the robotic hand below the natural one (Palmar Middle Central, PMC) (Figure 3B). In the PMC configuration, the misalignment of the robotic hand introduces an additional gravity torque that may lead to annoying rotations of the human arm and a major force requested to the subject. The same happens for the DDC configuration due to the distance of the robotic hand with respect to the natural one. Another aspect that is worth considering is that the robotic hand in the PMC configuration can hide the object to grasp forcing the user to move head and trunk to better see the item.

### 2.2. Experimental Tasks

**Figure 4:**
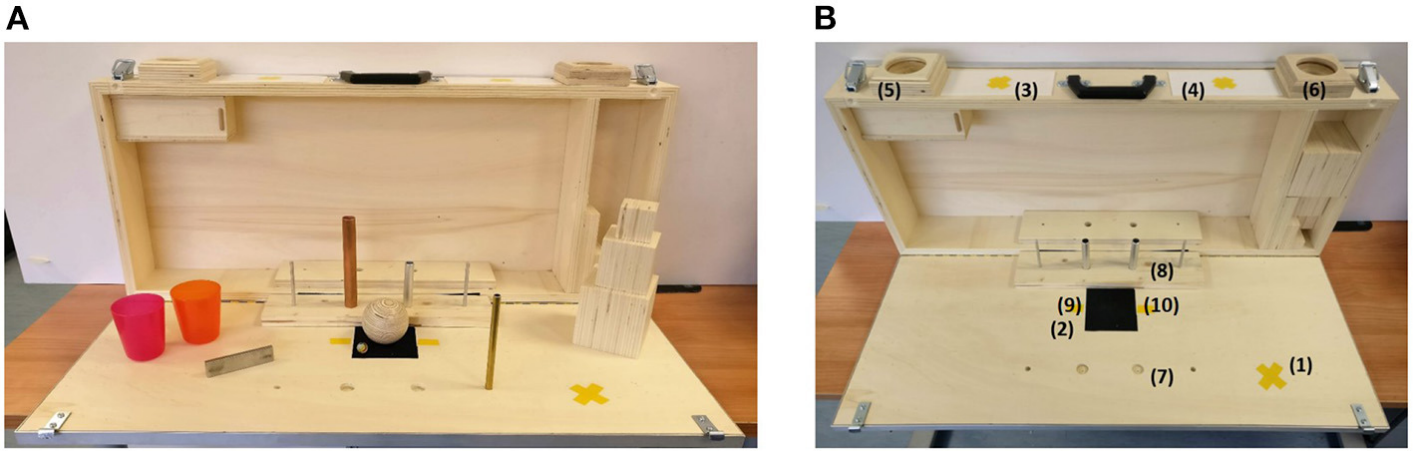
**(A)** Shows the kit of items for the Action Research Arm Test, while **(B)** the ARAT case. In particular, (1) is the hand starting position; (2) is the object starting position; the target position for the contralateral side is in (3) while for the ipsilateral side in (4); (5) and (6) are the target positions for the ball and the marble in the contralateral side and ipsilateral side, respectively; (7) and (8) are the starting hole and the ending peg for the tube tasks; (9) and (10) are the positions of the glasses for the pouring task.

Subjects were asked to perform a modified version of the Action Research Arm Test (mARAT). This test (Lyle, 1981) is traditionally used in clinic to assess upper extremity performance in post-stroke patients. It involves the manipulation of objects differing in size, weight and shape (as shown in Figure 4A) starting from the same predefined position [(2) in Figure 4B]. For this study, the test has been modified with respect to the standard version. The gross movements (e.g., place hand behind head or to mouth) were not executed due to the encumbrance of the system. In addition, some items and activities were removed (e.g., the biggest wood cube, the washer and the ball bearing), since not compatible with the dimension and grasping capacity of the robotic hand. In details, the executed tasks, in order of execution, were the following:

- Lifting objects from a starting position [(2) in Figure 4B] to a higher one: in order 3 wood cubes (2.5, 5, 7.5 cm^3^), a sharpening stone (10 × 2.5 × 1 cm), a ball (7.5 cm diameter), and a marble (1.5 cm diameter);
- Moving two metal tubes (2.25 × 11.5 and 1.0 × 16.0 cm) from a hole [(7) in Figure 4B] to a peg [(8) in Figure 4B];
- Pouring the content of a glass [positioned in (9) in Figure 4B] into another one [positioned in (10) in Figure 4B].

**Figure 5:**
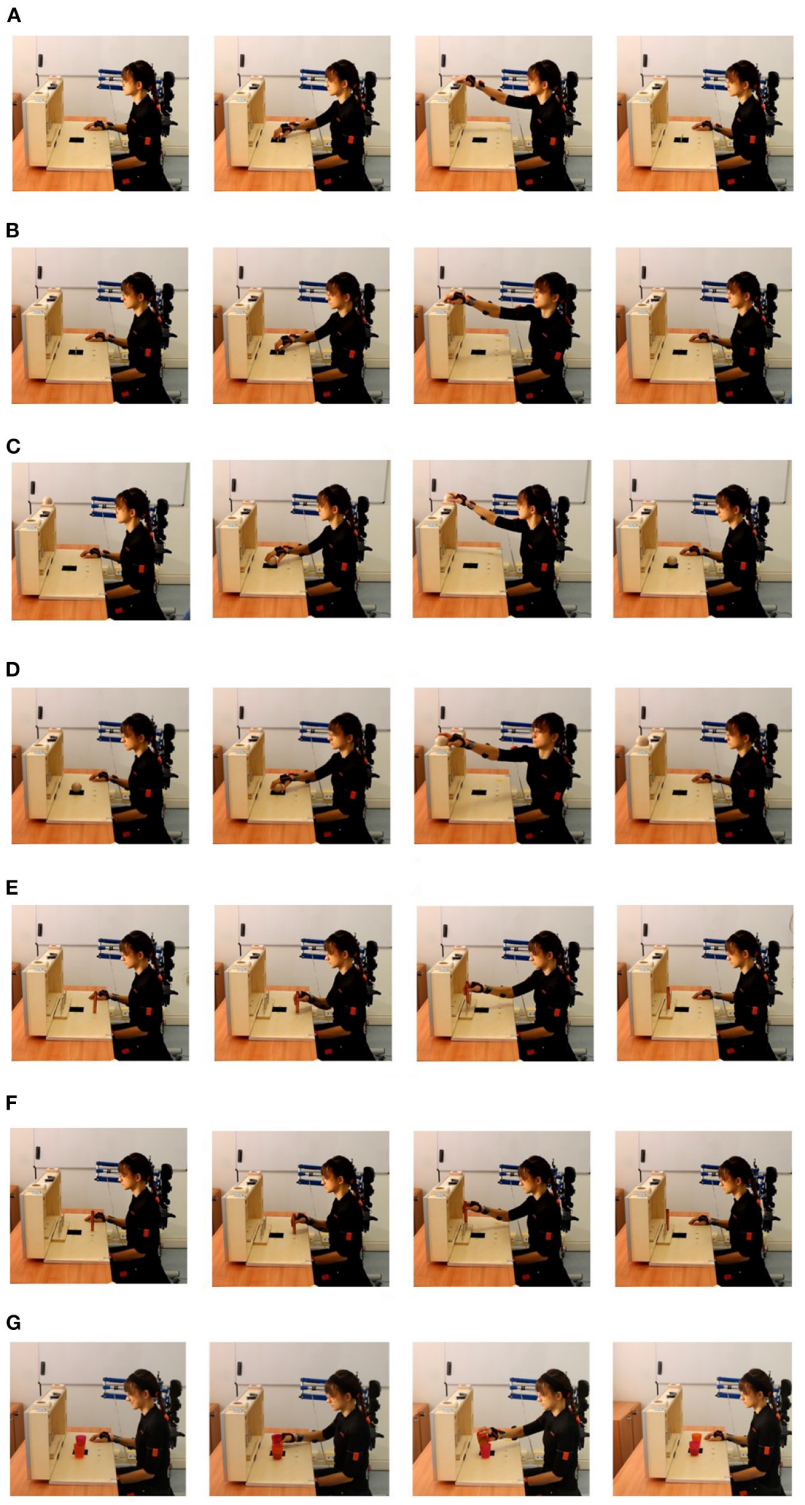
Photo sequences of the tasks. **(A)** Shows the lifting of the sharpening stone in the ipsilateral side, **(B)** the lifting of the sharpening stone in the contralateral side, **(C)** the lifting of the ball in the ipsilateral side, **(D)** the lifting of the ball in the contralateral side, **(E)** the moving of the tube in the ipsilateral side, **(F)** the moving of the tube in the contralateral side, and **(G)** the pouring of the content of a glass into another.

Photo sequences showing some of the task executions are reported in Figure 5. The single task was considered accomplished when the object reached the target and the right hand came back to the starting position [(1) in Figure 4B]. In order to explore the whole reachable workspace, both the ipsilateral and the contralateral side with respect to the robotic system placement were explored. The objects were first moved from the starting position to the ipsilateral target (see Figures 5A,B,E) and then to the contralateral target (see Figures 5B,D,F. Each task was repeated three times in a row, positioning every time the object at the starting point.

### 2.3. Experimental Procedure

**Figure 6:**
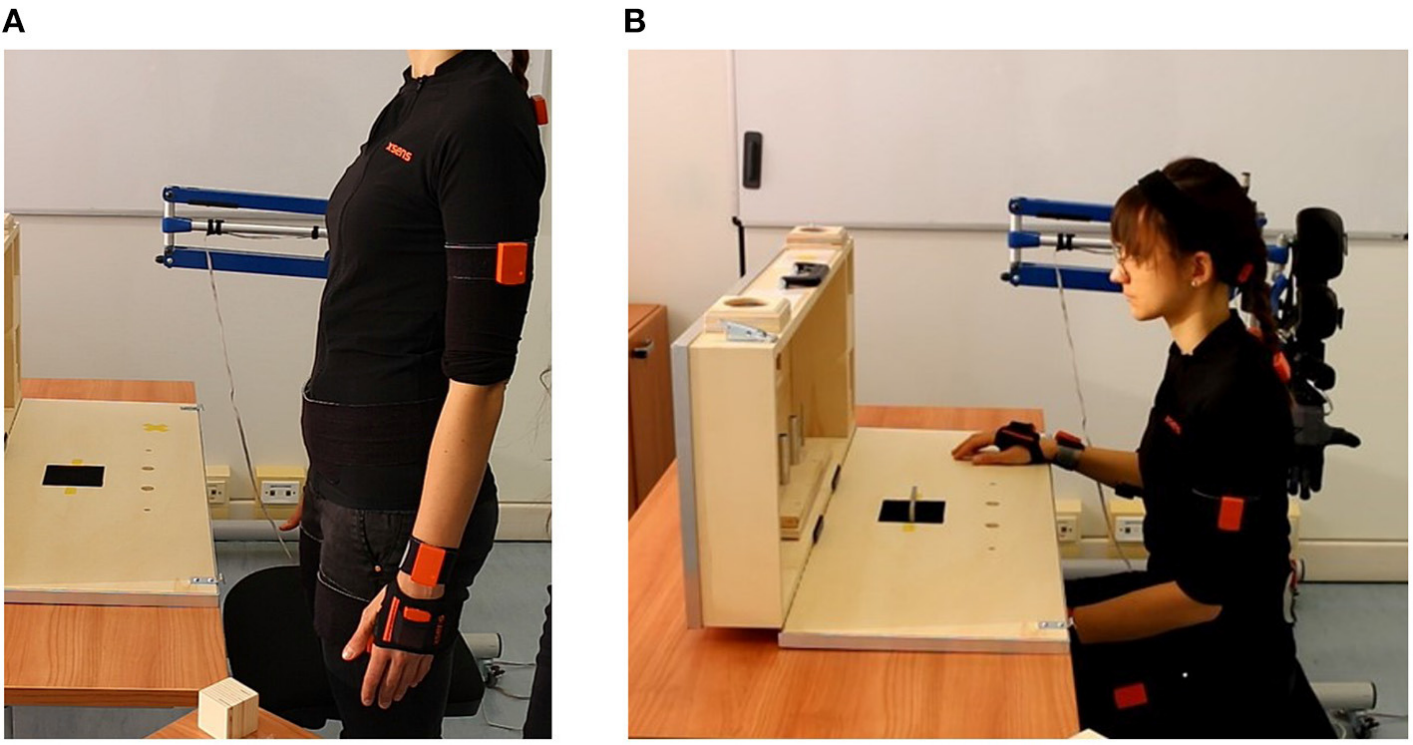
**(A)** Shows the N-pose for the Xsens calibration while **(B)** shows the starting position of the experiment.

First, the experiment was introduced to the subject, describing him/her both the SHX system, including the data acquiring systems, and the aim of the study. Then, all the sensors were placed on the subject's body, as shown in Figure 2B. IMU sensors placements were done as suggested by the Xsens User's Manual. Muscles for the EMG electrodes, instead, were manually identified by the experimenter. In addition, body measurements of the subjects (e.g., legs and arms length) were inserted into the Xsens software to reconstruct the virtual body model and estimate the joint movements. A phase of calibration was then executed. To calibrate the Xsens system, he/she was asked to stay still in a stand position called N-pose (Figure 6A). He/she had to stand upright, feet parallel, back straight, arm straight alongside the body (vertically), thumbs and face forwards. To assess the starting position (shown in Figure 6B), the height of the chair was set so that the subject could touch the table with his/her fingers, with the elbow flexed at 90°, while the distance from the table had to allow the subject touching the high back of the ARAT case. During the execution of the test with the SHX system, the robotic hand was positioned at the same starting position of the right natural hand and both the height and the distance were re-setted to fit the new configuration adopted. These calibration procedures were conducted before each experimental condition tested (natural hand, DDC, and PMC). The subject executed the tasks first with his/her own hand, then using the SHX system in the two configurations cited. We asked them to keep the left arm at rest during execution since no bimanual tasks needed to be performed. Before starting the experiment, all the subjects had some minutes of training and during configuration changes they were given a few minutes to rest and the calibrations were repeated. In these experiments, we did not ask the subject to execute the tasks as fast as possible to avoid the rush to influence movements. We decided not to evaluate the performances of execution since this aspect has been already investigated in Ciullo et al. (2018b) obtaining that the execution with the DDC configuration resulted longer in most of the tasks.

### 2.4. Data Analysis

To quantitatively evaluate compensatory movements, a comparison between the tasks execution with the natural hand and the execution with the SHX system was performed, considering the first as reference of optimal behavior. Moreover, a comparison between the two different configurations of the SHX system was performed. After the acquisition, the kinematic data and the EMG signals obtained were exported in Matlab 2018b and different indices were computed (see Table 1). Taking inspiration from literature (Carey et al., 2008; Metzger et al., 2012; Hussaini et al., 2017), the Range of Motion (RoM) of the joints was calculated as the difference between the maximum and the minimum measured angles (Equation 1).

(1) $$\begin{array}{r} RoM=max\left(angle\right)-min\left(angle\right) \end{array}$$

**Table 1:** Variables calculated to evaluate the compensatory movement performed by the subject.

	Abduction/adduction
RoM of the shoulder	Rotation
	Flexion/extension
RoM of the elbow	Flexion/extension
RoM of the wrist	pronation/supination
	Abduction/adduction
RoM of the trunk	Rotation
	Flexion/extension
	Abduction/adduction
RoM of the head	Rotation
	Flexion/extension
Accuracy index	Intra-subject accuracy
	Inter-subject accuracy
Efficiency index	Intra-subject efficiency
	Inter-subject efficiency
RMS value of the EMG signals	

The movements considered are the abduction/adduction, the flexion/extension and the rotation of the right shoulder, head and trunk, the pronation/supination of the wrist and the flexion/extension of the right elbow. For the wrist, the other degrees of freedom were not considered since they were limited by the human-arm interface of the SHX system.

An accuracy index and an efficiency index were computed, as described in de los Reyes-Guzmán et al. (2017), to give a measure of the differences between the natural hand trajectory and the hand path length exploited by the subjects during the tasks execution with and without the robotic system. In particular, two different comparisons were conducted: *inter-subjects* and *intra-subjects*. The main difference consists of the way evaluating the reference hand trajectory (*s*_*ref*_) (see Equations 2, 3):

(2) $$\begin{array}{r} s_{ref}^{inter}=\overset{N}{\underset{i=1}{\sum }}\frac{s_{\text{i}}}{N} \end{array}$$

where *N* is the number of subjects, and *s*_i_ represents the single subject trajectory obtained during the execution of the test with the natural hand. For intra-subject comparison:

(3) $$\begin{array}{r} s_{ref}^{intra}=\underset{s_{\text{j}}}{\arg\min}|s_{\text{j}}-s_{\text{mean}}| \end{array}$$

where *j* = *1,2,3* is the number of the trial and *s*_mean_ is the mean hand trajectory among the three repetitions obtained during the execution of the test with the natural hand. Trajectories of different lengths have been resampled with the Matlab function *interp1* (Matlab 2018b) with a linear interpolation to allow comparisons.

Then, the accuracy index (*A*) was defined as the product of three different terms (Equation 4) and then normalized (*A*_norm_ in Equation 5).

(4) $$\begin{array}{r} A=\alpha \cdot \rho \cdot BN \end{array}$$

(5) $$\begin{array}{r} A_{\text{norm}}=\frac{A}{A_{\text{ref}}}\cdot 100 \end{array}$$

The term α is a sigmoid function (see de los Reyes-Guzmán et al., 2017) depending on the mean distance between the reference hand trajectory (inter or intra) and the analyzed one, the term ρ is the Pearson correlation coefficient between these two trajectories and, lastly, the term *BN* was defined as the percentage of the analyzed trajectory within a dispersion band around the reference hand trajectory considered acceptable. This dispersion band for the inter-subject comparison (*DB*^*inter*^) was calculated as:

(6) $$\begin{array}{r} DB^{inter}=s_{ref}^{inter}\pm 2\cdot std \end{array}$$

where *std* is the standard deviation around the reference trajectory. For the intra-subject comparison, the dispersion band (*DB*^*intra*^) was:

(7) $$\begin{array}{r} DB^{intra}=s_{ref}^{intra}\pm 2\cdot \overset{3}{\underset{i=1}{\sum }}\frac{s_{ref}^{intra}-s_{\text{i}}}{3} \end{array}$$

The constant (c = 2) multiplying the standard deviation was chosen following the same procedure of de los Reyes-Guzmán et al. (2017). In particular, the dipersion band DB was calculated using c = 1 and c = 1.5 obtaining very low values of the accuracy and efficiency indices (<10) and using c = 2.5 and c = 3 obtaining too high values (in some cases >100). So the good solution was to choose the intermediate value that was c = 2.

The efficiency index (*E*_norm_) was defined by normalizing the hand path length *p* with respect to the reference one *p*_ref_ (inter or intra):

(8) $$\begin{array}{r} E_{\text{norm}}=\frac{p}{p_{\text{ref}}}\cdot 100 \end{array}$$

In de los Reyes-Guzmán et al. (2017), authors inverted the equation (see Equation 9) because the hand path length of patients was always longer than the healthy one.

(9) $$\begin{array}{r} E_{\text{norm}}=\frac{p_{\text{ref}}}{p}\cdot 100 \end{array}$$

In our case, the hand path length without the SHX system was not always shorter than the one with the system so both Equations (8) and (9) were used.

For each robotic configuration we considered the mean values, over the three trials, of the accuracy and efficiency indices. These indices were computed also for the natural hand in order to evaluate both how much the single subject behavior was different from the mean of all the subjects and how different were the trajectories during the three repetitions of the task.

EMG signals were exported in Matlab 2018b and band-pass filtered (10–500 Hz; Buttherworth 9th order), note that this has also the effect of removing the signal mean value. The signals obtained were rectified and normalized. In literature, the normalization is often performed on the maximum contraction level. Since our task caused a really small effort, it was not convenient to normalize in this way, so the maximum value obtained all the tasks was used. Finally the envelope was extracted by filtering the signal with a Butterworth low-pass filter of the 5th order with a cut-off frequency of 10 Hz. To evaluate the muscles activation, the RMS value was calculated for each subject and for each task as the mean value between the ones obtained for the three repetitions in the interval in which the muscle was activated.

For the statistical analysis, a one-sample Kolmogorov-Smirnov test was performed to assess if the data came from a standard normal distribution. This hypothesis was rejected so a non-parametric version of classical one-way ANOVA was necessary. Kruskal-Wallis test was used to statistically compared the configurations with the hand and between each other. The significance level for all statistical comparisons was set at *p* < 0.05. The comparison was performed, for all the variables and for each task, between the three different configurations considering the values obtained from the 11 subjects.

In the end, to simplify the comparison, the tasks executed were divided in five functional groups, as shown in Table 2. This division was done since it was noticed that, due to the similarity of movement, tasks of the same group showed similar values for the variables extracted. Then for each task, the median value of all the variables and indices was obtained among the values of all the subjects.

**Table 2:** Functional division of the tasks.

**Group 1—Contralateral lifting tasks**	
Task 1	Lifting of the ball
Task 3	Lifting of the smaller cube
Task 5	Lifting of the middle cube
Task 7	Lifting of the bigger cube
Task 9	Lifting of the marble
Task 11	Lifting of the sharpening stone
**Group 2—Ipsilateral lifting tasks**	
Task 2	Lifting of the ball
Task 4	Lifting of the smaller cube
Task 6	Lifting of the middle cube
Task 8	Lifting of the bigger cube
Task 10	Lifting of the marble
Task 12	Lifting of the sharpening stone
**Pouring task**	
Task 13	Pouring the content of a glass
**Group 4—Contralateral tube tasks**	
Task 14	Lifting of the bigger tube
Task 16	Lifting of the smaller tube
**Group 5—Ipsilateral tube tasks**	
Task 15	Lifting of the bigger tube
Task 17	Lifting of the smaller tube

## 3. Results

### 3.1. Kinematic Analysis

**Figure 7:**
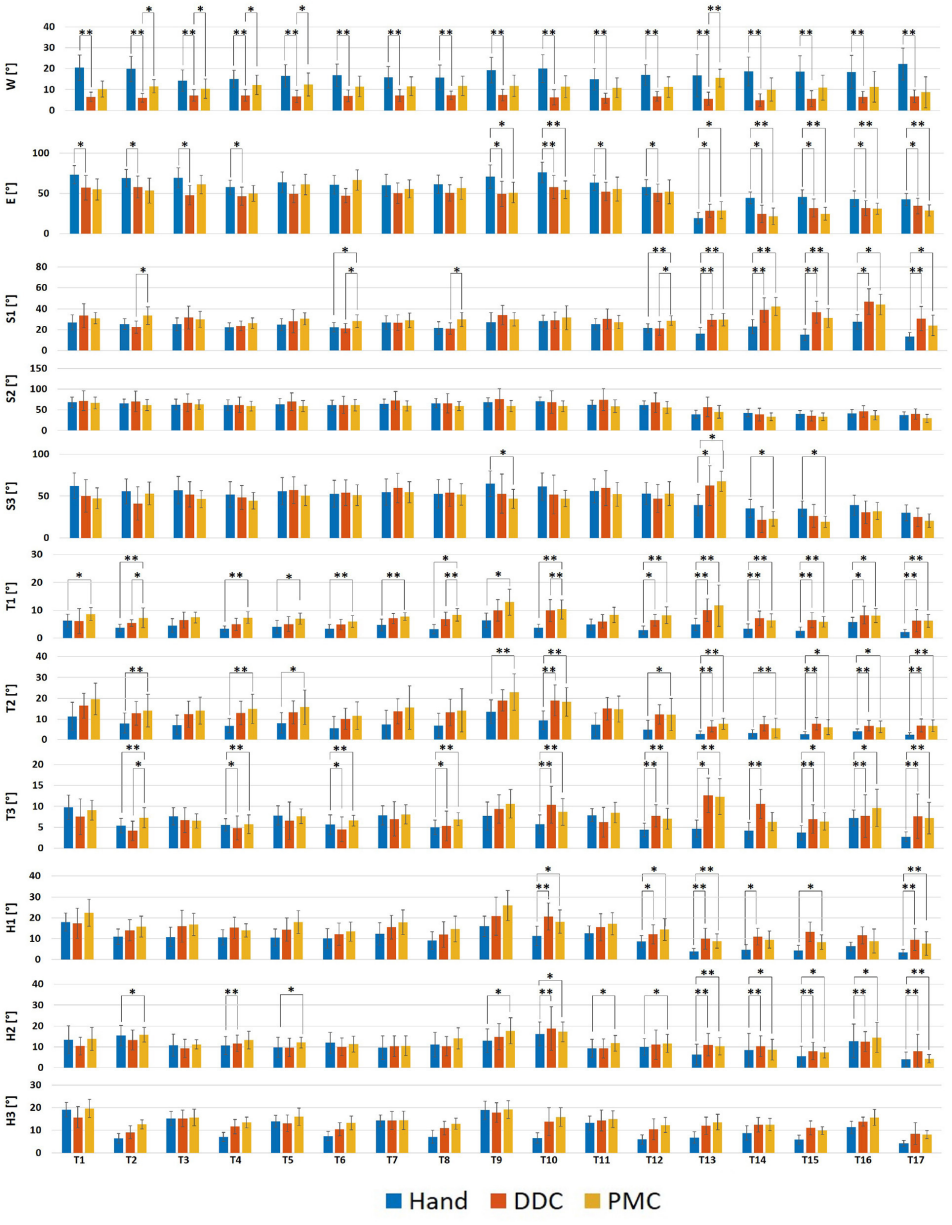
Bar plots showing the median values and the standard deviations upon all the subjects of the RoM obtained during the execution of the tasks in the three configurations tested. S1, Shoulder abduction/adduction; S2, Shoulder flexion/extension; S3, Shoulder rotation; E, Elbow flexion/extension; W, Wrist pronation/supination; T1, Trunk abduction/adduction; T2, Trunk flexion/extension; T3, Trunk rotation; H1, Head abduction/adduction; H2, Head flexion/extension; H3, Head rotation. *p < 0.05, **p < 0.01.

Figure 7 shows the median values of the Ranges of Motion obtained from the execution with and without the robotic hand.

#### 3.1.1. RoM of the Wrist

For the pronation/supination, statistically significant differences have been obtained in tasks 1, 2, 7, 8, 9, 10, 12, 13, 14, 15, 16, 17 (more than 10° lower), 3, 4, 5, 6, and 11 (<10° lower) for the DDC configuration with respect to the free execution, in none of the tasks for the PMC configuration with respect to the free execution and in tasks 2, 3, 4, 5, 12, and 13 (<10° lower) for the DDC configuration with respect to the PMC configuration.

#### 3.1.2. RoM of the Elbow

For the flexion/extension, statistically significant differences have been obtained in tasks 1, 2, 4, 10, 11, 14, 15, 16 (more than 10° lower), 3, 9 (more than 20° lower), 12, 17 (more than 5° lower), and 13 (more than 5° higher) for the DDC configuration with respect to the free execution, in tasks 9, 16, 17 (more than 10° lower), 10, 14, 15, (more than 20° lower), and 13 (more than 5° higher) for the PMC configuration with respect to the free execution and in none of the tasks for the DDC configuration with respect to the PMC configuration.

#### 3.1.3. RoM of the Shoulder

For the abduction/adduction, statistically significant differences have been obtained in tasks 13, 14, 16, 17 (more than 10° higher), and 15 (more than 20° higher) for the DDC configuration with respect to the free execution, in tasks 6, 12 (<10° higher), 13, 14, 15, 16, and 17 (more than 10° higher) for the PMC configuration with respect to the free execution and in tasks 2 (more than 10° higher), 6, 8, and 12 (<10° higher) for the DDC configuration with respect to the PMC configuration. For the flexion/extension, no statistically significant differences have been obtained. For the rotation, statistically significant differences have been obtained only in task 13 (more than 20° higher) for the DDC configuration with respect to the free execution, in tasks 9, 14, 15 (more than 10° lower), 13 (more than 20° higher), and 17 (more than 5° lower) for the PMC configuration with respect to the free execution and in none of the tasks for the DDC configuration with respect to the PMC configuration.

#### 3.1.4. RoM of the Trunk

For the abduction/adduction, statistically significant differences have been obtained in tasks 8, 10, 11, 12, 13, 14, 15, 16, and 17 (<10° higher) for the DDC configuration with respect to the free execution, in tasks 1, 2, 4, 5, 6, 7, 8, 9, 10, 11, 12, 13, 14, 15, 16, and 17 (<10° higher) for the PMC configuration with respect to the free execution and only in task 2 (<10° lower) for the DDC configuration with respect to the PMC configuration. For the flexion/extension, statistically significant differences have been obtained in tasks 10, 13, 14, 15, 16, and 17 (<10° higher) for the DDC configuration with respect to the free execution, in tasks 2, 4, 5, 9, 10, 12, 13, 15, 16, and 17 (<10° higher) for the PMC configuration with respect to the free execution and in none of the tasks for the DDC configuration with respect to the PMC configuration. For the rotation, statistically significant differences have been obtained in tasks 4, 6, 8, 10, 11, 12, 13, 14, 15 and 17 (<10° higher) for the DDC configuration with respect to the free execution, in tasks 2, 4, 6, 8, 10, 11, 12, 13, 14, 15, 16, and 17 (<10° higher) for the PMC configuration with respect to the free execution and only in task 2 (<10° higher) for the DDC configuration with respect to the PMC configuration.

#### 3.1.5. RoM of the Head

For the abduction/adduction, statistically significant differences have been obtained in tasks 10, 12, 13, 14, 15, and 17 (<10° higher) for the DDC configuration with respect to the free execution, in tasks 10, 12, 13, and 17 (<10° higher) for the PMC configuration with respect to the free execution and in none of the tasks for the DDC configuration with respect to the PMC configuration. For the flexion/extension, statistically significant differences have been obtained in tasks 4, 10, 13, 14, 15, 16, and 17 (<10° higher) for the DDC configuration with respect to the free execution, in tasks 2, 5, 9, 10, 11, 12, 13, 14, 15, 16, and 17 (<10° higher) for the PMC configuration with respect to the free execution and in none of the tasks for the DDC configuration with respect to the PMC configuration. For the rotation, no statistically significant differences have been obtained.

#### 3.1.6. Accuracy Index

**Figure 8:**
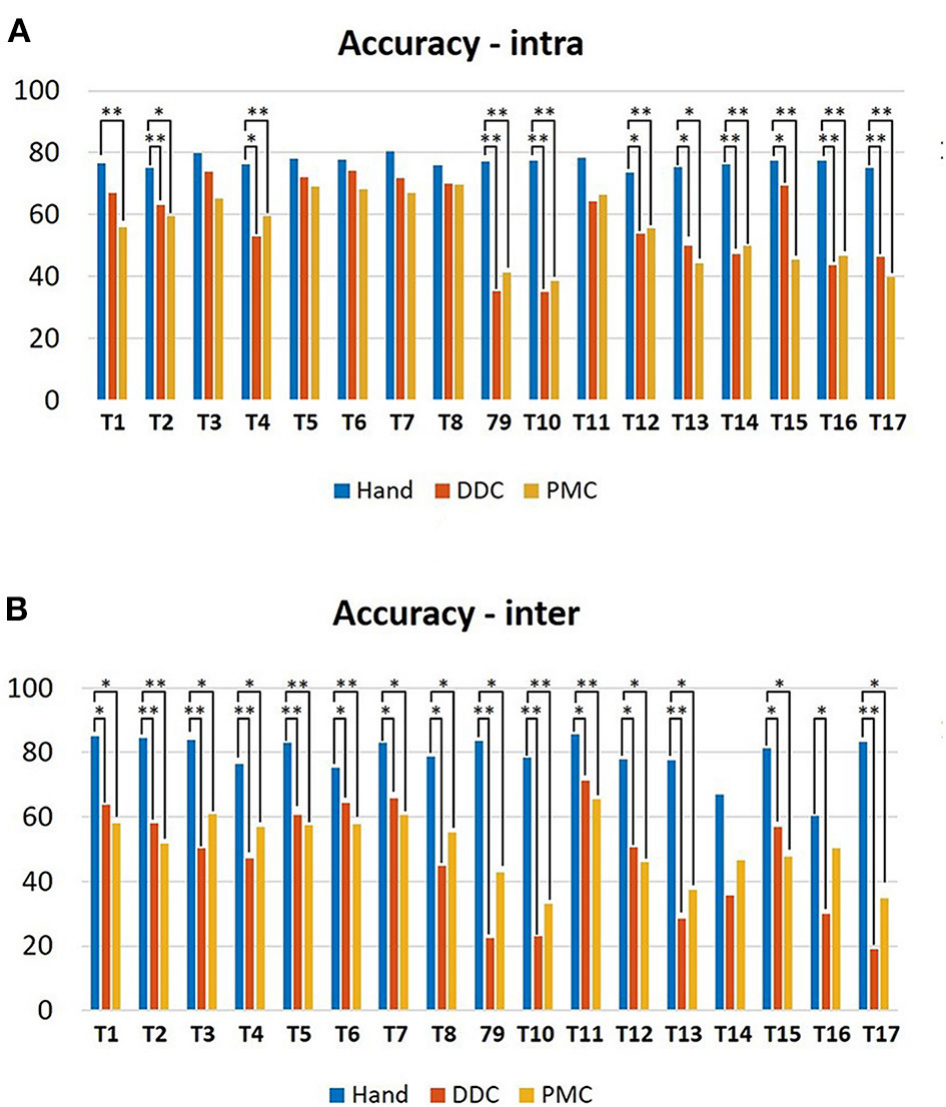
Bar plots showing the median values and the standard deviations upon all the subjects of the **(A)** intra-subject accuracy and **(B)** inter-subjects accuracy indices obtained during the execution of the tasks in the three configurations tested. *p < 0.05, **p < 0.01.

Figure 8 shows the median values of the (A) intra-subject and (B) inter-subjects accuracy index obtained from the execution with and without the robotic hand. For the intra-subject index, statistically significant differences have been obtained in tasks 2, 4, 9, 10, 12, 13, 14, 15, 16, and 17 for the DDC configuration with respect to the free execution, in tasks 1, 2, 4, 9, 10, 12, 13, 14, 15, 16, and 17 for the PMC configuration with respect to the free execution and in none of the tasks for the DDC configuration with respect to the PMC configuration. For the execution with the natural hand the values are in the range 73–81%, for the DDC configuration the values are lower than 50% in six tasks and lower than 75% for the other tasks while for the PMC configuration the values are lower than 50% in seven tasks and lower than 70% for the other tasks. For the inter-subject index, statistically significant differences have been obtained in tasks 1, 2, 3, 4, 5, 6, 7, 8, 9, 10, 11, 12, 13, 15, 16, and 17 for the DDC configuration with respect to the free execution, in tasks 1, 2, 3, 4, 5, 6, 7, 8, 9, 10, 11, 12, 13, 15, and 17 for the PMC configuration with respect to the free execution and in none of the tasks for the DDC configuration with respect to the PMC configuration. For the free execution the values are in the range 60–86%, for the DDC configuration the values are lower than 50% in eight tasks and lower than 72% for the other tasks while for the PMC configuration the values are lower than 50% in seven tasks and lower than 66% for the other tasks.

#### 3.1.7. Efficiency Index

**Figure 9:**
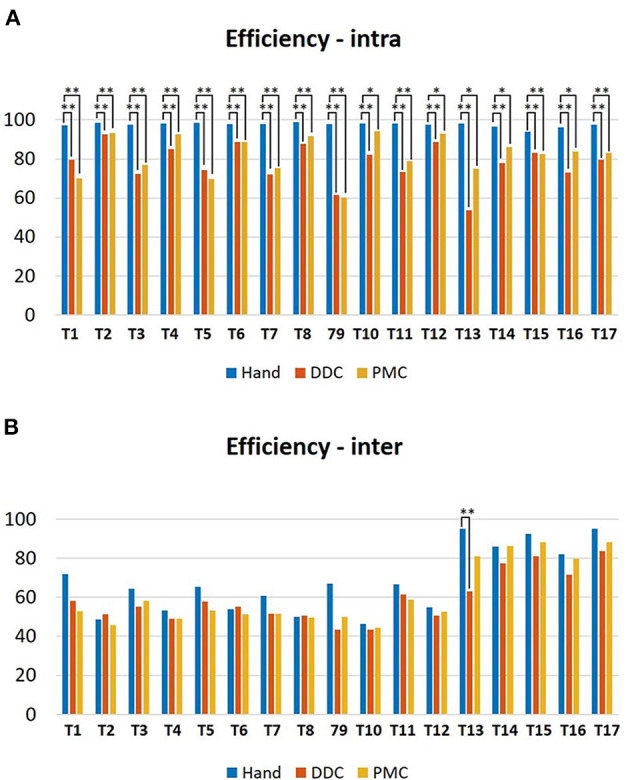
Bar plots showing the median values and the standard deviations upon all the subjects of the **(A)** intra-subject efficiency and **(B)** inter-subjects efficiency indices obtained during the execution of the tasks in the three configurations tested. *p < 0.05 **p < 0.01.

Figure 9 shows the median values of the (A) intra-subject and (B) inter-subjects efficiency index obtained from the execution with and without the robotic hand. For the intra-subject index, statistically significant differences have been obtained in all the tasks for the DDC configuration with respect to the free execution, in all the tasks for the PMC configuration with respect to the free execution and in none of the tasks for the DDC configuration with respect to the PMC configuration. For the free execution the values are in the range 93–99%, for the DDC configuration the values are in the range 53–93% while for the PMC configuration the values are in the range 60–95%. For the inter-subject index, statistically significant differences have been obtained only in task 13 for the DDC configuration with respect to the free execution, in none of the tasks for the PMC configuration with respect to the free execution and in none of the tasks for the DDC configuration with respect to the PMC configuration. For the free execution the values are lower than 50% in three tasks and lower than 95% for the other tasks, for the DDC configuration the values are lower than 50% in three tasks and lower than 84% for the other tasks while for the PMC configuration the values are lower than 50% in four tasks and lower than 89% for the other tasks.

### 3.2. EMG Signal Analysis

The execution of the tasks with the robotic system was always longer than the one with the natural hand, resulting in a longer activation period of the muscles. However, the values of the RMS resulted very similar between the three executions of the tasks. For the clarity of the results, only the RMS values of the deltoid, the trapezius and the triceps were reported in Supplementary Table S6 since only for this muscles some statistical differences have been found. In particular, they have been found for the deltoid between the natural hand and the DDC configuration in tasks 10 and 13, between the natural hand and the PMC configuration in tasks 1, 2, 3, 4, 10, 11, 12, 13, 15, and between the two configurations in tasks 1, 2, for the trapezius between the natural hand and the PMC configuration in task 6 and for the triceps between the natural hand and the DDC configuration in task 13. It is also interesting to analyse the fact that the muscle activity resulting from the natural hand shows always a relaxing period between the different repetitions when the hand came back to the starting position while during the execution with the SHX system the muscle activity is higher also in this phase (example in **Figure 11**).

## 4. Discussion

Most of the supernumerary robotic systems presented in literature have been specifically designed for augmenting workers' abilities in industrial applications (Llorens-Bonilla et al., 2012; Ciullo et al., 2018a). Most recently, the use of this technology has been also proposed for impaired assistance (Hussain et al., 2016). This can be an interesting opportunity for subjects with a permanent disability due to neuromuscular diseases, like post-stroke subjects, or injuries. In this scenario, the use of a supernumerary robotic hand can compensate for the missing functionalities by acting in charge of the natural impaired one (Ciullo et al., 2020). However, some considerations and analyses need to be done for safety in using this novel robot-assisted approach. Differently from other assistive robotic systems (Wu et al., 2013; Nordin et al., 2014; Grimm et al., 2016), where variations on movement patterns are mainly due to the subject's impairments, in supernumerary robotic hand such alteration can be introduced by the encumbrance of the additional robotic hand itself. To verify the presence and estimate the size of these compensatory movements, with this work a quantitative analysis has been conducted involving healthy subjects.

According to the kinematic results, we observed that the use of the apparatus reduces the range of motion of the wrist, elbow and shoulder, while it increases the range of the trunk and head movements. Regarding the shoulder joint, for the tasks of groups 1 and 2 (12 tasks in total), statistically significant differences have been found only for the abduction/adduction in 3/12 tasks for the DDC configuration and in 2/12 tasks for the PMC configuration. This shows that, for these tasks involving only grasping of objects, the shoulder movements were similar during the execution with and without the robotic system. However, a different situation can be seen in the pouring task (task 13) and in the lifting of the tubes (task 13, 14, 15, 16, and 17, so 5 in total) which required a major precision, forcing the subject to use more this joint. Indeed, for these tasks, statistically significant differences have been found for the abduction/adduction in 5/5 tasks for the DDC configuration and in 5/5 tasks for the PMC configuration, while for the flexion/extension in 3/5 tasks for the DDC configuration and in 1/5 tasks for the PMC configuration. Regarding the elbow joint, the RoM obtained during the execution with the robotic hand resulted lower with respect to the natural execution for all the tasks. This can be attributed to the presence of the gravity compensator helping the subject to move the arm without exploiting the elbow movement. The only task in which the RoM of the elbow was higher was task 13 (pouring). This is in line with the fact that, as previously said, this task also required a larger movement of the shoulder. Concerning the wrist pronation/supination, the RoM during the execution with the natural hand was higher for all the tasks. This was due to the fact that using the robotic system, the natural hand was fixed for grasping the handle controller, thus limiting the wrist movements. However, statistically significant differences have been obtained only for the DDC configuration. This aspect may prove that the execution with the PMC configuration was very similar to the execution with the natural hand. In this configuration, the robotic hand is below the natural one. This reduce the visual feedback, since the object is hidden by the robotic hand, requesting a major wrist pronation/supination. The RoM obtained from the trunk and the head movements are higher during the execution with the robotic hand, thus indicating that subjects used these movements to compensate the reduced exploitation of the arm joints. Also for trunk and head joints, the biggest differences are shown for the pouring task, the lifting of the marble and the smaller tube. This could be due both to the fact that these tasks required more dexterity, and because of the occlusion of the robotic hand pushing the subject to move the head and the trunk to better look at the object (Ciullo et al., 2018b). This was more evident with the PMC configuration for which, as already said, most of the subjects asserted that the object was hidden by the system. To solve this problem, a haptic feedback system could be added as proposed by Schofield et al. (2014) and Svensson et al. (2017).

**Figure 10:**
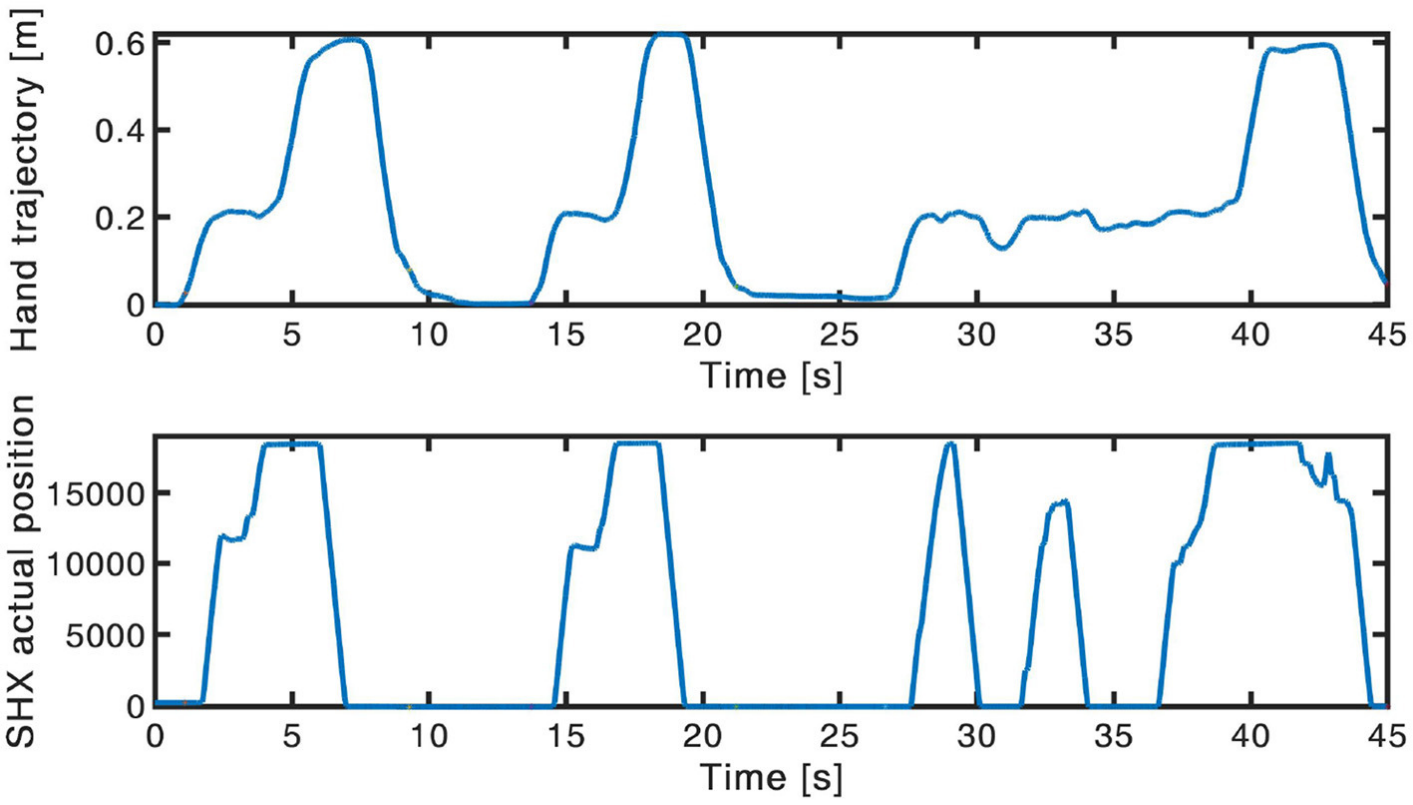
Example of hand trajectory exploited (up) and of the consequent SHX position (down) during the lifting of the marble in the contralateral side. In the third repetition the subject needed more attempts to accomplish the task, thus resulting in a more irregular trajectory.

Considering the accuracy and efficiency indices, the values were lower in the tasks requiring more dexterity (e.g., lifting of the marble and of the smallest tube). Moreover, in some cases, subjects needed more attempts to accomplish these tasks, thus resulting in more irregular hand trajectories (example in Figure 10). Nevertheless, also the values obtained during the executions with the natural hand were low (average 79.11% for the inter-subject accuracy and 67.79% for the inter-subject efficiency), reflecting an high variability between the subjects to accomplish the same task. This variability can explain the very low values (average 46.59% for the inter-subject accuracy in the DDC configuration, 50.76% for the inter-subject accuracy in the PMC configuration, 59.04% for the inter-subject efficiency in the DDC configuration, and 61.18% for the inter-subject efficiency in the PMC configuration) obtained for the two configurations of the SHX. In fact, it can be caused not only by the encumbrance of the robotic system but also by the difference in the strategy and trajectory used by different subjects.

**Figure 11:**
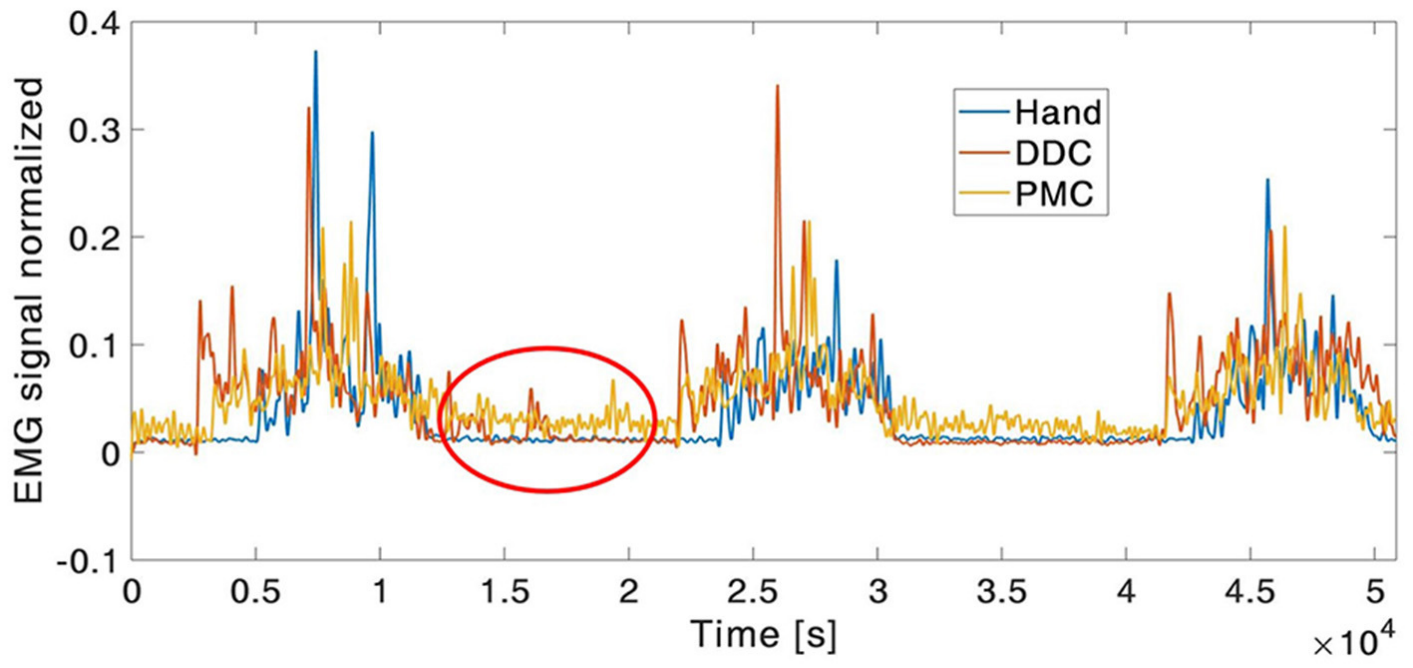
Example of EMG envelope signal of the wrist flexor. In the red circle, it can be noticed the presence of muscle activity also during the relaxing period between trial during the execution with robotic system.

For what concerns the muscles activity, the values of the RMS resulted similar between the three conditions and few statistically significant differences have been found. This could be justified by the fact that all the tasks proposed were quite short and easy to be executed for healthy subjects, and all the items had a light weight (the heaviest was <250 g). None of the subjects experienced evident level of fatigue and no evident differences among the natural hand and the robotic configurations were found. However, the execution with the robotic system was always longer than the one with the natural hand, resulting in a longer activation period of the muscles. Moreover, the muscle activity resulting from the natural hand shows always a relaxing period between the three repetitions of the tasks, when the hand come back to the starting position. During the execution with the SHX system the muscle activity was present also in this phase (see Figure 11). This was more evident for the wrist muscles in the DDC configuration. Two main reasons could justify this evidence: first the subject contracted the wrist muscles to maintain the position and hold the handle. The other reason is that, as already said, even if the subject is helped by the gravity compensator, during the use of the robotic system, the misalignment and the distance of the robotic hand, with respect to the subject's arm, introduces an additional gravity torque. This situation leads to annoying rotations of the human arm and a major force requested to the subject. The same problem was highlighted by stroke patients, using this human-arm interface during previous pilot studies. To counteract these effects, a new prototype of the human-harm interface may be developed with reduced weight and encumbrance and with the addition of a counter-mass.

For what concerns the comparison between the two robotic configurations no relevant differences have been obtained in the values of the indices considered so they can be selected in accordance to the subjects' preference or need. However, it was noticed that, as said in section 2.1, the annoying rotations caused by the misalignment and the distance of the robotic hand requested higher movements exploited to accomplish the task. This was true mostly for the DDC configuration. In particular, in the tube tasks in which the object were closer to the subjects trunk RoM resulted higher with respect to the other two executions. Also the problem of occlusion caused by the position of the hand in the PMC configuration was reported by subjects. Larger movement of the head have been reported during the execution with this configuration, in particular in tasks of group 1 and 2 involving the grasping of sobjects.

Although the absence of impaired subjects may represent a limit for this work, the normal condition of the involved ones ensures that any movement variations is mainly related to the system design. Another subject-related factor that may alter the movement execution, inducing compensation with trunk and head movements, can be the muscular fatigue. However, data analysis on the EMG signals have reported no differences on muscle activities during the whole experimental procedure, excluding in this way the fatigue as possible cause for movement compensations. For this reason, we did not consider muscle synergies even though they can provide interesting outcomes. Due to the ease of the tasks, very low variance among the different synergies would have been obtained. Another limit of this study could be the lack of a metric considering all the joints together, such as kinematic synergies. The huge amount of data acquired and the selected indices provide enough information for our analysis but future works will be designed to analyze the correlation between joints and include additional and more general indices.

## 5. Conclusion

This work provides a detailed description of the compensatory movements exploited by subjects using a supernumerary robotic hand for upper limb assistance. No relevant differences have been found between the two configurations tested so they can be selected in accordance to the subjects preference or need. By comparing the joints movement during the use of the robotic system with respect to the free execution, it has also been demonstrated that, the SHX system can be useful to reduce the stress on the wrist, elbow and shoulder joints, since the RoM exploited was very similar or decreased in the majority of the tasks. However, the use of the trunk and of the head increased. Moreover, from the EMG signals analysis, the muscles activity resulted very similar during the use of the system, thus demonstrating that this device is not detrimental from the point of view of the muscular fatigue. This work suggest that the system may be used as assistive device without causing an over-use of the arm joints and also opens the way to clinical trials with patients. Results may help to upgrade the system with a more comfortable and suitable human-arm interface to avoid occlusion of objects and larger movements of trunk and head. Future work will be oriented to the evaluation of the compensatory movements also with post-stroke patients.

## Data Availability Statement

The original contributions presented in the study are included in the article/Supplementary Material, further inquiries can be directed to the corresponding author/s.

## Ethics Statement

The studies involving human participants were reviewed and approved by Institutional Review Board of the University of Pisa. The patients/participants provided their written informed consent to participate in this study. Written informed consent was obtained from the individual(s) for the publication of any potentially identifiable images or data included in this article.

## Author Contributions

MR designed the experimental protocol and drafted the manuscript. ASC participated in the design of the experimental setup and revised the manuscript. MGC and GG supervised the engineering process and data analysis. AB supervised the research.

## Conflict of Interest

The authors declare that the research was conducted in the absence of any commercial or financial relationships that could be construed as a potential conflict of interest.

## Footnotes

^1^All raw data acquired are accessible on the open access repository, Hand Corpus.

## References

[B1] Ada L. Canning C. Carr J. Kilbreath S. Shepherd R. (1994). Task-specific training of reaching and manipulation. Adv. Psychol. 105, 239–265. 10.1016/S0166-4115(08)61281-9

[B2] Carey S. L. Highsmith M. J. Maitland M. E. Dubey R. V. (2008). Compensatory movements of transradial prosthesis users during common tasks. Clin. Biomech. 23, 1128–1135. 10.1016/j.clinbiomech.2008.05.008 18675497

[B3] Cirstea M. Levin M. F. (2000). Compensatory strategies for reaching in stroke. Brain 123, 940–953. 10.1093/brain/123.5.940 10775539

[B4] Ciullo A. S. Catalano M. G. Bicchi A. Ajoudani A. (2018a). A supernumerary soft robotic hand-arm system for improving worker ergonomics, in International Symposium on Wearable Robotics (Pisa: Springer), 520–524. 10.1007/978-3-030-01887-0_101

[B5] Ciullo A. S. Felici F. Catalano M. G. Grioli G. Ajoudani A. Bicchi A. (2018b). Analytical and experimental analysis for position optimization of a grasp assistance supernumerary robotic hand. IEEE Robot. Autom. Lett. 3, 4305–4312. 10.1109/LRA.2018.2864357

[B6] Ciullo A. S. Veerbeek J. M. Temperli E. Luft A. R. Tonis F. J. Haarman C. J.. (2020). A novel soft robotic supernumerary hand for severely affected stroke patients. IEEE Trans. Neural Syst. Rehabil. Eng. 28, 1168–1177. 10.1109/TNSRE.2020.2984717 32248115

[B7] de los Reyes-Guzmán A. Dimbwadyo-Terrer I. Pérez-Nombela S. Monasterio-Huelin F. Torricelli D. Pons J. L.. (2017). Novel kinematic indices for quantifying upper limb ability and dexterity after cervical spinal cord injury. Med. Biol. Eng. Comput. 55, 833–844. 10.1007/s11517-016-1555-0 27544674

[B8] Della Santina C. Piazza C. Gasparri G. M. Bonilla M. Catalano M. G. Grioli G. (2017). The quest for natural machine motion: An open platform to fast-prototyping articulated soft robots. IEEE Robot. Autom. Mag. 24, 48–56. 10.1109/MRA.2016.2636366

[B9] Grimm F. Naros G. Gharabaghi A. (2016). Compensation or restoration: closed-loop feedback of movement quality for assisted reach-to-grasp exercises with a multi-joint arm exoskeleton. Front. Neurosci. 10:280 10.3389/fnins.2016.00280 27445655 PMC4914560

[B10] Hussain I. Salvietti G. Spagnoletti G. Prattichizzo D. (2016). The soft-sixthfinger: a wearable EMG controlled robotic extra-finger for grasp compensation in chronic stroke patients. IEEE Robot. Autom. Lett. 1, 1000–1006. 10.1109/LRA.2016.2530793

[B11] Hussaini A. Zinck A. Kyberd P. (2017). Categorization of compensatory motions in transradial myoelectric prosthesis users. Prosthet. Orthot. Int. 41, 286–293. 10.1177/0309364616660248 27473642

[B12] Levin M. F. Kleim J. A. Wolf S. L. (2009). What do motor recovery and compensation mean in patients following stroke? Neurorehabil. Neural Repair 23, 313–319. 10.1177/1545968308328727 19118128

[B13] Llorens-Bonilla B. Parietti F. Asada H. (2012). Demonstration-based control of supernumerary robotic limbs, in RSJ International Conference on Intelligent Robots and Systems (IROS), 2012 (Vilamoura: IEEE), 7–12. 10.1109/IROS.2012.6386055

[B14] Lyle R. C. (1981). A performance test for assessment of upper limb function in physical rehabilitation treatment and research. Int. J. Rehabil. Res. 4, 483–492. 10.1097/00004356-198112000-00001 7333761

[B15] Maciejasz P. Eschweiler J. Gerlach-Hahn K. Jansen-Troy A. Leonhardt S. (2014). A survey on robotic devices for upper limb rehabilitation. J. Neuroeng. Rehabil. 11:3. 10.1186/1743-0003-11-3 24401110 PMC4029785

[B16] Major M. J. Stine R. L. Heckathorne C. W. Fatone S. Gard S. A. (2014). Comparison of range-of-motion and variability in upper body movements between transradial prosthesis users and able-bodied controls when executing goal-oriented tasks. J. Neuroeng. Rehabil. 11:132. 10.1186/1743-0003-11-132 25192744 PMC4164738

[B17] Metzger A. J. Dromerick A. W. Holley R. J. Lum P. S. (2012). Characterization of compensatory trunk movements during prosthetic upper limb reaching tasks. Archiv. Phys. Med. Rehabil. 93, 2029–2034. 10.1016/j.apmr.2012.03.011 22449551

[B18] Michaelsen S. M. Jacobs S. Roby-Brami A. Levin M. F. (2004). Compensation for distal impairments of grasping in adults with hemiparesis. Exp. Brain Res. 157, 162–173. 10.1007/s00221-004-1829-x 14985899

[B19] Mondiale de la Santé O. Organization W. H. (2001). International Classification of Functioning, Disability and Health: ICF. Geneva: World Health Organization.

[B20] Mozaffarian D. Benjamin E. J. Go A. S. Arnett D. K. Blaha M. J. Cushman M. (2015). Executive summary: heart disease and stroke statistics—2015 update: a report from the american heart association. Circulation 131, 434–441. 10.1161/CIR.0000000000000157 26811276

[B21] Nordin N. Xie S. Q. Wünsche B. (2014). Assessment of movement quality in robot-assisted upper limb rehabilitation after stroke: a review. J. Neuroeng. Rehabil. 11:137. 10.1186/1743-0003-11-137 25217124 PMC4180322

[B22] Parietti F. Asada H. H. (2017). Independent, voluntary control of extra robotic limbs, in 2017 IEEE International Conference on Robotics and Automation (ICRA) (Marina Bay Sands: IEEE), 5954–5961. 10.1109/ICRA.2017.7989702

[B23] Roby-Brami A. Feydy A. Combeaud M. Biryukova E. Bussel B. Levin M. (2003). Motor compensation and recovery for reaching in stroke patients. Acta Neurol. Scand. 107, 369–381. 10.1034/j.1600-0404.2003.00021.x 12713530

[B24] Santello M. Flanders M. Soechting J. F. (1998). Postural hand synergies for tool use. J. Neurosci. 18, 10105–10115. 10.1523/JNEUROSCI.18-23-10105.1998 9822764 PMC6793309

[B25] Schofield J. S. Evans K. R. Carey J. P. Hebert J. S. (2014). Applications of sensory feedback in motorized upper extremity prosthesis: a review. Expert Rev. Med. Dev. 11, 499–511. 10.1586/17434440.2014.929496 24928327

[B26] Svensson P. Wijk U. Björkman A. Antfolk C. (2017). A review of invasive and non-invasive sensory feedback in upper limb prostheses. Expert Rev. Med. Dev. 14, 439–447. 10.1080/17434440.2017.1332989 28532184

[B27] Wade D. T. (1992). Measurement in neurological rehabilitation. Curr. Opin. Neurol. Neurosurg. 5, 682–686. 1392142

[B28] Wu C. Yang C. Chen M. Lin K. Wu L. (2013). Unilateral versus bilateral robot-assisted rehabilitation on arm-trunk control and functions post stroke: a randomized controlled trial. J. Neuroeng. Rehabil. 10:35. 10.1186/1743-0003-10-35 23587106 PMC3640972

