# Supplementary material for: Analysis of Compensatory Movements Using a Supernumerary Robotic Hand for Upper Limb Assistance
Source: Front Robot AI. 2020 Dec 17;7:587759. doi: 10.3389/frobt.2020.587759 (PMC7805947; doi:10.3389/frobt.2020.587759)
Supplement: Supplementary file 1 [file Data_Sheet_1.PDF]

# *Supplementary Material*

## 1 SUPPLEMENTARY TABLES

**Table S1.** Median values upon all the subjects of the RoM obtained during the execution of the tasks of group 1 in the three configurations tested. S1=Shoulder abduction/adduction, S2=Shoulder flexion/extension, S3=Shoulder rotation, E=Elbow flexion/extension, W=Wrist pronation/supination, T1=Trunk abduction/adduction, T2=Trunk flexion/extension, T3=Trunk rotation, H1=Head abduction/adduction, H2=Head flexion/extension, H3=Head rotation

| | **Task 1** | | | **Task 3** | | | **Task 5** | | | **Task 7** | | | **Task 9** | | | **Task 11** | | |
|---|---|---|---|---|---|---|---|---|---|---|---|---|---|---|---|---|---|---|
| | **Hand** | **DDC** | **PMC** | **Hand** | **DDC** | **PMC** | **Hand** | **DDC** | **PMC** | **Hand** | **DDC** | **PMC** | **Hand** | **DDC** | **PMC** | **Hand** | **DDC** | **PMC** |
| **W** | 20.46 | 6.45 | 10.21 | 14.20 | 7.15 | 10.43 | 16.51 | 6.69 | 12.37 | 15.93 | 7.03 | 11.59 | 19.23 | 7.35 | 11.78 | 14.92 | 5.87 | 10.79 |
| **E** | 73.23 | 57.32 | 55.12 | 69.30 | 47.82 | 61.05 | 64.01 | 49.51 | 61.20 | 60.31 | 50.26 | 55.65 | 70.91 | 49.44 | 51.08 | 63.26 | 52.26 | 55.50 |
| **S1** | 26.95 | 33.48 | 30.99 | 25.17 | 31.60 | 29.88 | 24.88 | 27.98 | 30.46 | 26.76 | 26.69 | 28.85 | 27.15 | 33.79 | 30.02 | 25.09 | 30.13 | 27.11 |
| **S2** | 67.91 | 71.83 | 66.68 | 62.77 | 67.04 | 62.93 | 63.29 | 69.79 | 59.30 | 64.30 | 72.08 | 59.81 | 68.41 | 75.50 | 59.12 | 62.66 | 74.56 | 58.63 |
| **S3** | 62.36 | 50.35 | 47.40 | 56.95 | 51.96 | 46.37 | 55.63 | 57.13 | 50.73 | 54.79 | 59.64 | 54.60 | 64.67 | 52.80 | 46.75 | 56.19 | 59.60 | 52.21 |
| **T1** | 6.30 | 6.15 | 8.66 | 4.54 | 6.49 | 7.43 | 4.12 | 5.06 | 6.99 | 4.80 | 7.07 | 7.74 | 6.36 | 9.92 | 12.95 | 4.88 | 6.05 | 8.36 |
| **T2** | 11.32 | 16.45 | 19.68 | 7.23 | 12.40 | 14.11 | 8.15 | 13.26 | 15.74 | 7.41 | 13.84 | 15.56 | 13.61 | 18.99 | 22.97 | 7.28 | 15.06 | 14.83 |
| **T3** | 19.10 | 15.72 | 19.65 | 15.12 | 15.18 | 15.63 | 13.96 | 13.01 | 15.91 | 14.19 | 14.26 | 14.37 | 18.81 | 17.73 | 19.12 | 13.12 | 14.31 | 14.86 |
| **H1** | 9.79 | 7.51 | 9.08 | 7.63 | 6.69 | 6.53 | 7.82 | 6.57 | 7.59 | 7.85 | 6.97 | 8.06 | 7.67 | 9.38 | 10.62 | 7.91 | 6.20 | 8.45 |
| **H2** | 18.00 | 17.34 | 22.47 | 10.91 | 15.99 | 16.76 | 10.58 | 14.33 | 18.01 | 12.30 | 15.46 | 17.84 | 15.94 | 20.82 | 25.89 | 12.70 | 15.46 | 17.12 |
| **H3** | 13.45 | 10.42 | 13.76 | 10.79 | 9.25 | 11.23 | 9.84 | 9.73 | 12.07 | 9.65 | 10.29 | 10.55 | 12.94 | 14.85 | 17.68 | 9.37 | 9.26 | 11.78 |

**Table S2.** Median values upon all the subjects of the RoM obtained during the execution of the tasks of group 2 in the three configurations tested. S1=Shoulder abduction/adduction, S2=Shoulder flexion/extension, S3=Shoulder rotation, E=Elbow flexion/extension, W=Wrist pronation/supination, T1=Trunk abduction/adduction, T2=Trunk flexion/extension, T3=Trunk rotation, H1=Head abduction/adduction, H2=Head flexion/extension, H3=Head rotation

| | Task 2 | | | Task 4 | | | Task 6 | | | Task 8 | | | Task 10 | | | Task 12 | | |
|---|---|---|---|---|---|---|---|---|---|---|---|---|---|---|---|---|---|---|
| | **Hand** | **DDC** | **PMC** | **Hand** | **DDC** | **PMC** | **Hand** | **DDC** | **PMC** | **Hand** | **DDC** | **PMC** | **Hand** | **DDC** | **PMC** | **Hand** | **DDC** | **PMC** |
| **W** | 19.78 | 6.00 | 11.60 | 15.02 | 7.15 | 12.22 | 16.80 | 6.91 | 11.43 | 15.71 | 7.29 | 11.70 | 20.08 | 6.20 | 11.36 | 16.98 | 6.75 | 11.16 |
| **E** | 69.11 | 57.90 | 53.64 | 57.60 | 46.66 | 50.20 | 60.80 | 47.10 | 66.65 | 61.47 | 50.72 | 56.36 | 76.14 | 57.95 | 54.56 | 57.86 | 50.88 | 52.02 |
| **S1** | 25.38 | 22.43 | 33.44 | 22.39 | 23.61 | 26.34 | 22.27 | 21.33 | 28.15 | 21.68 | 21.05 | 29.77 | 28.15 | 28.93 | 31.37 | 21.52 | 21.34 | 28.70 |
| **S2** | 66.14 | 70.38 | 61.49 | 61.58 | 61.86 | 59.33 | 61.38 | 61.97 | 61.25 | 65.95 | 65.53 | 59.10 | 70.53 | 68.50 | 58.79 | 61.69 | 67.05 | 55.52 |
| **S3** | 55.57 | 41.04 | 52.91 | 51.83 | 48.34 | 44.43 | 52.66 | 53.90 | 51.09 | 52.62 | 53.77 | 51.99 | 61.18 | 51.85 | 46.94 | 53.01 | 47.01 | 52.90 |
| **T1** | 3.80 | 5.46 | 7.29 | 3.33 | 4.96 | 7.38 | 3.32 | 4.88 | 6.01 | 3.21 | 6.92 | 8.34 | 3.72 | 9.93 | 10.30 | 2.87 | 6.45 | 8.28 |
| **T2** | 8.05 | 12.77 | 14.09 | 6.76 | 12.98 | 14.92 | 5.64 | 10.17 | 11.63 | 6.93 | 13.23 | 14.13 | 9.52 | 19.01 | 18.25 | 5.03 | 12.30 | 12.09 |
| **T3** | 6.46 | 9.07 | 12.58 | 7.01 | 11.57 | 13.41 | 7.41 | 10.35 | 13.11 | 7.07 | 10.94 | 12.92 | 6.56 | 13.83 | 15.78 | 5.91 | 10.42 | 12.31 |
| **H1** | 5.41 | 4.13 | 7.30 | 5.56 | 4.82 | 5.71 | 5.62 | 4.49 | 6.59 | 4.97 | 5.27 | 6.91 | 5.70 | 10.38 | 8.67 | 4.37 | 7.68 | 7.01 |
| **H2** | 10.99 | 14.03 | 15.84 | 10.67 | 15.26 | 13.96 | 10.18 | 12.13 | 13.41 | 9.14 | 12.07 | 14.63 | 11.28 | 20.57 | 18.19 | 8.67 | 12.10 | 14.42 |
| **H3** | 15.39 | 13.30 | 15.75 | 10.72 | 11.69 | 13.23 | 11.93 | 10.05 | 11.31 | 11.07 | 10.31 | 14.06 | 16.20 | 18.77 | 17.24 | 9.97 | 11.08 | 11.66 |

**Table S3.** Median values upon all the subjects of the RoM obtained during the execution of the pouring task (task 13) and tasks of group 4 and 5 in the three configurations tested.
S1=Shoulder abduction/adduction, S2=Shoulder flexion/extension, S3=Shoulder rotation, E=Elbow flexion/extension, W=Wrist pronation/supination, T1=Trunk abduction/adduction, T2=Trunk flexion/extension, T3=Trunk rotation, H1=Head abduction/adduction, H2=Head flexion/extension, H3=Head rotation

| | **Task 13** | | | **Task 14** | | | **Task 15** | | | **Task 16** | | | **Task 17** | | |
|---|---|---|---|---|---|---|---|---|---|---|---|---|---|---|---|
| | **Hand** | **DDC** | **PMC** | **Hand** | **DDC** | **PMC** | **Hand** | **DDC** | **PMC** | **Hand** | **DDC** | **PMC** | **Hand** | **DDC** | **PMC** |
| **W** | 16.64 | 5.56 | 15.52 | 18.75 | 4.86 | 9.97 | 18.52 | 5.59 | 10.93 | 18.34 | 6.40 | 11.31 | 22.18 | 6.70 | 8.69 |
| **E** | 19.37 | 28.39 | 29.00 | 44.50 | 24.72 | 21.59 | 45.84 | 32.09 | 24.80 | 43.26 | 31.72 | 31.06 | 42.63 | 34.38 | 28.90 |
| **S1** | 16.25 | 29.12 | 29.60 | 22.84 | 38.75 | 42.22 | 15.16 | 36.74 | 31.35 | 27.57 | 46.75 | 44.02 | 13.29 | 30.55 | 24.05 |
| **S2** | 39.14 | 56.91 | 45.15 | 42.20 | 38.83 | 33.22 | 39.60 | 35.20 | 33.16 | 41.40 | 46.27 | 36.70 | 37.50 | 39.51 | 29.44 |
| **S3** | 39.11 | 62.46 | 67.66 | 35.24 | 21.62 | 22.90 | 34.96 | 26.13 | 19.16 | 38.83 | 30.50 | 32.06 | 29.77 | 24.78 | 20.34 |
| **T1** | 4.90 | 10.05 | 11.69 | 3.42 | 7.10 | 6.37 | 2.58 | 6.44 | 5.85 | 5.69 | 8.29 | 8.14 | 2.10 | 6.20 | 6.19 |
| **T2** | 2.74 | 6.53 | 7.80 | 3.12 | 7.70 | 5.67 | 2.58 | 7.82 | 6.08 | 3.97 | 6.81 | 6.21 | 2.36 | 6.95 | 6.75 |
| **T3** | 6.79 | 11.93 | 13.54 | 8.79 | 12.41 | 12.49 | 5.84 | 11.06 | 9.80 | 11.28 | 13.82 | 15.62 | 4.15 | 8.46 | 8.04 |
| **H1** | 4.66 | 12.62 | 12.30 | 4.15 | 10.57 | 6.32 | 3.70 | 6.96 | 6.39 | 7.19 | 7.66 | 9.65 | 2.75 | 7.61 | 7.18 |
| **H2** | 3.86 | 9.97 | 8.92 | 4.71 | 10.97 | 9.56 | 4.28 | 13.30 | 8.36 | 6.30 | 11.66 | 8.90 | 3.45 | 9.57 | 7.67 |
| **H3** | 6.34 | 11.05 | 10.34 | 8.54 | 10.31 | 8.68 | 5.52 | 8.02 | 7.30 | 12.80 | 12.52 | 14.51 | 4.05 | 8.06 | 4.37 |

**Table S4.** Intra-subject accuracy and efficiency indices. *p<0.05 **p<0.01

| | Accuracy index | | | p-value | | | Efficiency index | | | p-value | | |
|---|---|---|---|---|---|---|---|---|---|---|---|---|
| | Hand | DDC | PMC | Hand vs DDC | Hand vs PMC | DDC vs PMC | Hand | DDC | PMC | Hand vs DDC | Hand vs PMC | DDC vs PMC |
| **Task 1** | 76,57 | 66,86 | 56,04 | 0.15 | 0.02* | 0.69 | 97,23 | 79,43 | 70,18 | ** | ** | 0.94 |
| **Task 2** | 75,17 | 63,03 | 59,43 | 0.04* | ** | 0.51 | 98,64 | 92,72 | 93,25 | ** | ** | 0.92 |
| **Task 3** | 79,96 | 73,84 | 65,27 | 0.51 | 0.19 | 0.8 | 97,54 | 72,23 | 76,98 | ** | ** | 0.96 |
| **Task 4** | 76,29 | 52,88 | 59,62 | ** | 0.02* | 0.75 | 98,19 | 85,09 | 92,62 | ** | ** | 0.67 |
| **Task 5** | 78,05 | 72,05 | 69,22 | 0.20 | 0.31 | 0.96 | 98,35 | 74,28 | 69,79 | ** | ** | 1 |
| **Task 6** | 77,73 | 74,10 | 68,09 | 0.94 | 0.38 | 0.58 | 97,98 | 88,61 | 88,73 | ** | ** | 0.95 |
| **Task 7** | 80,46 | 71,84 | 67,05 | 0.50 | 0.38 | 0.98 | 97,86 | 72,09 | 75,41 | ** | ** | 1 |
| **Task 8** | 75,87 | 70,12 | 69,76 | 0,47 | 0.34 | 0.97 | 98,90 | 87,60 | 91,52 | ** | ** | 0.83 |
| **Task 9** | 77,22 | 35,40 | 41,37 | ** | ** | 0.99 | 98,02 | 61,47 | 60,26 | ** | ** | 1 |
| **Task 10** | 77,46 | 34,86 | 38,47 | ** | ** | 0.86 | 98,07 | 82,10 | 94,24 | ** | 0.04* | 0.09 |
| **Task 11** | 78,49 | 64,19 | 66,36 | 0.2 | 0.08 | 0.90 | 98,23 | 73,26 | 78,72 | ** | ** | 0.98 |
| **Task 12** | 73,52 | 53,83 | 55,67 | 0,01* | ** | 0.89 | 97,66 | 88,72 | 93,03 | ** | 0.01* | 0.32 |
| **Task 13** | 75,27 | 49,91 | 44,20 | 0.01* | 0.01* | 1 | 98,15 | 53,48 | 74,99 | ** | 0.008* | 0.20 |
| **Task 14** | 76,21 | 47,30 | 49,97 | ** | ** | 0.99 | 96,60 | 77,88 | 86,17 | ** | 0.02* | 0.10 |
| **Task 15** | 77,56 | 69,39 | 45,52 | 0.02* | ** | 0.50 | 93,88 | 83,04 | 82,49 | ** | ** | 0.11 |
| **Task 16** | 77,43 | 43,78 | 46,57 | ** | ** | 0.90 | 96,31 | 73,08 | 83,70 | ** | 0.007* | 0.72 |
| **Task 17** | 75,20 | 46,38 | 39,89 | ** | ** | 0.97 | 97,54 | 79,41 | 82,98 | ** | ** | 0.96 |

**Table S5.** Inter-subject accuracy and efficiency indices. *p<0.05 **p<0.01

| | **Accuracy index** | | | **p-value** | | | **Efficiency index** | | | **p-value** | | |
|---|---|---|---|---|---|---|---|---|---|---|---|---|
| | **Hand** | **DDC** | **PMC** | **Hand vs DDC** | **Hand vs PMC** | **DDC vs PMC** | **Hand** | **DDC** | **PMC** | **Hand vs DDC** | **Hand vs PMC** | **DDC vs PMC** |
| **Task 1** | 84,89 | 63,89 | 57,90 | 0,02* | 0,01* | 0,95 | 71,65 | 58,24 | 52,74 | 0,79 | 0,67 | 0,98 |
| **Task 2** | 84,32 | 58,17 | 51,86 | ** | ** | 0,62 | 48,49 | 51,35 | 45,81 | 0,65 | 0,45 | 0,09 |
| **Task 3** | 83,97 | 50,21 | 60,82 | ** | 0,01* | 0,88 | 64,19 | 55,12 | 57,93 | 0,91 | 0,83 | 0,99 |
| **Task 4** | 76,47 | 47,21 | 56,77 | ** | 0,03* | 0,24 | 53,18 | 49,08 | 49,05 | 0,68 | 0,57 | 0,98 |
| **Task 5** | 82,98 | 60,65 | 57,39 | ** | ** | 0,99 | 65,42 | 57,71 | 53,34 | 0,96 | 0,97 | 1,00 |
| **Task 6** | 75,33 | 64,28 | 57,86 | 0,01* | ** | 0,90 | 53,79 | 54,99 | 51,08 | 0,88 | 0,88 | 0,60 |
| **Task 7** | 82,87 | 65,66 | 60,67 | 0,04* | 0,03* | 0,99 | 60,61 | 51,64 | 51,49 | 0,60 | 0,73 | 0,97 |
| **Task 8** | 78,75 | 44,88 | 55,23 | 0,01* | 0,03* | 0,93 | 49,96 | 50,63 | 49,55 | 0,99 | 0,45 | 0,51 |
| **Task 9** | 83,56 | 22,43 | 42,72 | ** | 0,01* | 0,83 | 66,77 | 43,32 | 50,00 | 0,23 | 0,65 | 0,73 |
| **Task 10** | 78,39 | 22,98 | 33,10 | ** | ** | 0,49 | 46,45 | 43,24 | 44,51 | 0,43 | 0,53 | 0,99 |
| **Task 11** | 85,64 | 71,19 | 65,63 | 0,01* | ** | 0,97 | 66,66 | 61,49 | 58,82 | 0,80 | 0,93 | 0,96 |
| **Task 12** | 77,93 | 50,46 | 45,98 | 0,01* | 0,01* | 0,98 | 54,88 | 50,63 | 52,68 | 1,00 | 0,64 | 0,69 |
| **Task 13** | 77,69 | 28,50 | 37,39 | ** | 0,02* | 0,80 | 95,13 | 62,97 | 80,94 | ** | 0,06 | 0,15 |
| **Task 14** | 66,94 | 35,54 | 46,61 | 0,07 | 0,21 | 0,87 | 85,91 | 77,25 | 86,15 | 0,17 | 0,97 | 0,26 |
| **Task 15** | 81,42 | 56,97 | 47,69 | 0,02* | 0,01* | 0,98 | 92,39 | 81,09 | 88,19 | 0,09 | 0,54 | 0,54 |
| **Task 16** | 60,45 | 29,96 | 50,43 | 0,04* | 0,23 | 0,72 | 81,90 | 71,42 | 79,60 | 0,24 | 0,60 | 0,80 |
| **Task 17** | 83,27 | 19,05 | 34,88 | ** | 0,01* | 0,34 | 95,01 | 83,51 | 88,14 | 0,08 | 0,39 | 0,68 |

**Table S6.** RMS values obtained from the EMG signals of the deltoid, the trapezius and the triceps. *p<0.05 **p<0.01

| | Deltoid | | | Trapezius | | | Triceps | | |
|---|---|---|---|---|---|---|---|---|---|
| | **Hand** | **DDC** | **PMC** | **Hand** | **DDC** | **PMC** | **Hand** | **DDC** | **PMC** |
| **Task 1** | 0,1046 | 0,1084* | 0,0816* | 0,1075 | 0,1289 | 0,1064 | 0,1116 | 0,1187 | 0,0977 |
| **Task 2** | 0,1090 | 0,1060* | 0,0901* | 0,1190 | 0,1216 | 0,1009 | 0,1302 | 0,1244 | 0,1109 |
| **Task 3** | 0,1243 | 0,1041* | 0,0891 | 0,1183 | 0,1263 | 0,1024 | 0,1784 | 0,1274 | 0,1362 |
| **Task 4** | 0,1239 | 0,1038* | 0,0947 | 0,1219 | 0,1100 | 0,1107 | 0,1940 | 0,1278 | 0,1300 |
| **Task 5** | 0,1084 | 0,1207 | 0,0881 | 0,1204 | 0,1129 | 0,1093 | 0,1160 | 0,1333 | 0,1165 |
| **Task 6** | 0,1165 | 0,1214 | 0,0900 | 0,1155 | 0,1187* | 0,0938 | 0,1338 | 0,1156 | 0,1059 |
| **Task 7** | 0,1042 | 0,1070 | 0,0984 | 0,1129 | 0,1134 | 0,1195 | 0,1210 | 0,1125 | 0,1205 |
| **Task 8** | 0,1040 | 0,1048 | 0,0804 | 0,1152 | 0,1166 | 0,0987 | 0,1351 | 0,1254 | 0,1202 |
| **Task 9** | 0,1078 | 0,0939 | 0,0872 | 0,1024 | 0,1091 | 0,1040 | 0,1393 | 0,1115 | 0,1226 |
| **Task 10** | 0,1083** | 0,0831** | 0,0779 | 0,1209 | 0,1055 | 0,1033 | 0,1262 | 0,0950 | 0,0992 |
| **Task 11** | 0,1256 | 0,1141* | 0,0972 | 0,1078 | 0,1227 | 0,1087 | 0,1251 | 0,1232 | 0,1396 |
| **Task 12** | 0,1066 | 0,0958* | 0,0927 | 0,1243 | 0,1232 | 0,1016 | 0,1718 | 0,1310 | 0,1260 |
| **Task 13** | 0,1446** | 0,0797* | 0,0728 | 0,1208 | 0,1054 | 0,0957 | 0,1237 | 0,0844* | 0,0950 |
| **Task 14** | 0,1383 | 0,1112 | 0,1284 | 0,1111 | 0,0988 | 0,0951 | 0,1447 | 0,1456 | 0,1223 |
| **Task 15** | 0,1445 | 0,1322* | 0,1211 | 0,1278 | 0,1029 | 0,0969 | 0,1161 | 0,1424 | 0,1309 |
| **Task 16** | 0,1418 | 0,1330 | 0,1285 | 0,1162 | 0,1151 | 0,0970 | 0,1243 | 0,1373 | 0,1433 |
| **Task 17** | 0,1261 | 0,1314 | 0,1191 | 0,1146 | 0,1024 | 0,1009 | 0,1173 | 0,1459 | 0,1313 |
